# Reports from the Case Book of J. Junius Harris, M. D., and Horatio N. Hollifield, M. D. Tennille, Georgia

**Published:** 1855-09

**Authors:** 


ARTICLE VI.—*Reports from the Case Book of* J. JUNIUS HARRIS, M. D., and HORATIO N. HOLLIFIELD, M. D., of Tennille, Washington County, Ga.

*Editors Atlanta Medical and Surgical Journal:*

GENTLEMEN: Having recently received the "Prospectus" of a journal which you propose publishing in Atlanta, to be devoted to the interests of the Medical Sciences in the South, and having at heart a genuine desire that our noble profession may attain that dignity which it so well merits, but which, in the South, as yet, it cannot claim, we feel called upon, as members of that profession, to throw in our mite towards the accomplishment of that praiseworthy object. The truth is, and it should shame every Southern physician, we are too fond of building up northern schools, journals, &c., while our own are left to languish for the want of appropriate encouragement. We yield, with unbecoming docility, to the *ipse dixit* of some northern teacher of medicine, without once asking ourselves the highly important question, do the facts which he enunciates apply unconditionally to disease as it exists here? or are they not materially modified by climate or other operating causes? Oh, no! say we, that would be a work of supererogation; suffice it for us to know that professor somebody or somebody else said so—that's *law and gospel.*

It is against this condition of things that we wish to exercise our prerogative as independent, thinking, intelligent men, and if boldly "facing the music" can effect so desirable an end, to stimulate our brethren to a similar mode of thinking and acting. In the two cases, accounts of which we have appended, we do not expect to suggest anything new as to treatment. There are one or two points having a bearing upon the diagnosis, which are perhaps worthy of notice, especially to the young practitioner. It is true they are cases of not every day occurrence, and are happily illustrative of the power of medicine, aided by sound and discriminating judgment, over disease, and teach us how great a boon it is to the human race when in the proper hands. Our main object, however, is to set an example to the physicians of Georgia and of the South, viz: of giving to their brethren whatever of value they may acquire in the course of their practice. We would not be understood to intimate that we should tax the space of our journals with long and tedious accounts of all the cases which we may treat; but that in the practice of every physician some fact is established, or some truth illustrated, an account of which, with the attendant circumstances, would be of service to some, if not all, the members of the profession who might read it. Then let us determine that whenever anything of value occurs within our knowledge, as for instance the establishment of a fact in medicine, the illustration of an important truth in diagnosis, or the clearing up of a hidden point in pathology, it shall at once become the property of the profession; and in order to make it such, we will write out accounts of such items, and send them to the editors of our journals, periodicals, &c., to be published. By thus lending numerous rays of light to the several foci throughout our Southern country, brilliant suns will be formed, whose effulgence will shed lustre upon the most hidden mysteries, and such things as now constitute *opprobria medicorum*, will be converted into plain common sense truths.

CASE 1—*Stricture Urethra.*—On the 10th July we were consulted by T. C., ætas 30—temperament bilious—who stated that there was a slight discharge from the penis, and that the evacuations of the bladder were attended with a burning pain at the external urethral orifice. The idea of incipient gonorrhœa or of gleet at once suggested itself. In the course of our examinations, however, the following historical facts were ascertained. Seven years ago the patient, while making a journey on horseback, was attacked with gonorrhœa, but supposing it would soon disappear, from ignorance as to the nature of the disease, he neglected taking any of the usual remedies, and in accordance with his belief, but contrary to what might have been expected, all the symptoms disappeared in about six months, and he regarded himself as well; nor was there a return of symptoms until 1854, when the discharge and ardor urinæ again occurred, nor did the symptoms disappear until the patient was prostrated with what he termed rheumatism. He suffered from this attack for about six months, during which time he was unable to work, when the enlargement of the joints, pain, &c., passed off, and he resumed his occupation, which he continued until 1st of July, 1855, when the discharge and ardor urinæ again occurred; soon after which he asked for advice.

Suspicions of a stricture were at once entertained. An ordinary silver catheter was inserted, but was impeded in its course about an inch and a half from the external urethral orifice, and withdrawing the catheter, there was observed caught in its eyes an offensive matter. Bougies were at once substituted, and only the smallest size could be introduced, the withdrawal of which discovered the existence of another stricture about an inch lower, evidenced by the biting, grasping, sensation which was clearly perceptible.

Treatment was simple. A decoction of the leaves of Uva Ursi and a solution of Iodide of Potassium, five grains to the ounce of water, three times a day, and the introduction of gum elastic Bougies, every three or four days, commencing with the smaller size and gradually enlarging, until now the stricture is removed, the patient hearty, and regularly at work.

CASE NO. 2—*Inflamation of the Bladder.*—Early on the morning of the 10th July, we were called on to visit a woman, in our neighborhood, laboring under what we were informed was a severe attack of colic. There was great pain in the sacrum, and the abdomen was very sensitive to the touch; the skin hot and dry; the tongue coated, and the pulse weak though frequent. Small doses of morphia were administered, and the patient soon felt much easier. We left ten grains of Calomel to be given to the patient immediately, and ordered a strong decoction of Uva Ursi. The patient, however, continued to grow worse until evening, when we were again called. The symptoms were greatly aggravated; pain in the pelvis more severe; micturition was very frequent; the urine which was passed before our arrival, and which we examined, was mixed with a white, flaky matter, resembling mucus; and during our visit the urine voided contained blood, as well as mucus, and the passing of it caused the patient great pain, and was accompanied with continued straining; in fact, the agony of the patient was intense. We further noticed that the urine was of a dirty brown color and very offensive odor. We put her under the influence of Opium; continued the uva ursi as a drink, and applied opiate plasters to the parts where the pain was greatest. Under this treatment, the patient gradually improved, and is now in full health.

